# Effects of methanol and formic acid on human platelet aggregation

**DOI:** 10.1186/s12199-017-0687-7

**Published:** 2017-12-16

**Authors:** Mikio Marumo, Ichiro Wakabayashi

**Affiliations:** Department of Environmental and Preventive Medicine, Hyogo College of Medicine, Mukogawa-cho 1-1, Nishinomiya, Hyogo 663-8501 Japan

**Keywords:** Ca^2+^ channels, Capacitative Ca^2+^ entry, Ethanol, Formic acid, Methanol, Platelet aggregation

## Abstract

**Background:**

Although ethanol is known to inhibit platelet aggregation, the effects of another variant of alcohol, methanol, have not been reported. The purpose of this study was to determine whether methanol and its metabolite, formic acid, affect Ca^2+^ entry into and subsequent aggregation of platelets in vitro.

**Methods:**

Ca^2+^ entry into and aggregation of human platelets were measured by spectrofluorometry using Fura-2/AM as an indicator and the light transmission method, respectively.

**Results:**

Thrombin-induced platelet aggregation was significantly augmented by methanol at pharmacological concentrations (0.5–2%) in a concentration-dependent manner. Methanol at 2% significantly attenuated thapsigargin-induced platelet aggregation, which was not significantly affected by lower concentrations (0.5 and 1%) of methanol. Methanol (0.5–2%) did not significantly affect platelet aggregation induced by 1-oleoyl-2-acetyl-sn-glycerol (OAG), or Ca^2+^ entry into platelets induced by thrombin, thapsigargin, or OAG. Platelet aggregation induced by thrombin, thapsigargin, or OAG was significantly inhibited by formic acid at toxic concentrations (0.01% or higher). Ca^2+^ entry into platelets induced by thrombin or thapsigargin was also significantly inhibited by formic acid at 0.01% or higher, while that induced by OAG was not affected by formic acid at 0.005 and 0.01% and augmented by that at 0.02%.

**Conclusions:**

Methanol at pharmacological doses has diverse effects on platelet aggregation, depending on the aggregation stimuli, without affecting Ca^2+^ entry into platelets. Formic acid at toxic concentrations has an inhibitory action on platelets aggregation, which was partly explained by the reduction of Ca^2+^ entry into platelets.

## Background

Alcohol drinking is known to have diverse effects on the risk of cardiovascular disease that depend on the amount of ethanol consumed; the risk is reduced by moderate drinking but is increased by excessive drinking [1, 2]. The mechanism of the former beneficial effect is explained primarily by ethanol-induced increases in blood HDL cholesterol levels [3]. Attenuation of blood coagulability by drinking also lowers cardiovascular risk. Inhibition of platelet aggregation by ethanol [4, 5] is known to underlie the decreased blood coagulability in drinkers compared with nondrinkers, as is the decrease in blood levels of coagulation factors, including fibrinogen, in drinkers [6].

Methanol, another variant of alcohol, is used often in industry and is known to cause optic nerve injury and metabolic acidosis through the production of formic acid in vivo [7]. However, to the best of our knowledge, there have been no reports on effects of methanol and its metabolite, formic acid, on platelet function. Brain hemorrhage has been observed in 13.5% of patients with methanol intoxication [8]. Thus, there is a possibility of blood coagulation disorder caused by methanol intoxication.

Transplasmalemmal Ca^2+^ entry is a crucial step in the platelet activation process [9]. Our previous study demonstrated the diverse effects of ethanol on transplasmalemmal Ca^2+^ entry into the platelets through various pathways [10]. The purpose of the present study was to determine whether and how methanol and formic acid affect Ca^2+^ entry into and subsequent aggregation of platelets following different stimuli. For this purpose, we used three known platelet stimulants: thrombin, thapsigargin, and 1-oleoyl-2-acetyl-sn-glycerol (OAG).

## Methods

### Preparation of washed platelet suspension

Blood was obtained from healthy donors who had been medication-free for at least 10 days prior to the experiments. This study was approved by the Ethics Committee of Hyogo College of Medicine (No. 1799), and the experimental procedures were in accordance with the Helsinki Declaration. Blood (18 ml) was rapidly transferred to a plastic tube containing 2 ml of 3.2% sodium citrate and mixed. The blood was then centrifuged at 150×*g* for 10 min, and the supernatant was obtained as a platelet-rich plasma (PRP). PRP was subsequently mixed with 40 ml of Ca^2+^- and Mg^2+^-free Tyrode solution buffered by Hepes (NaCl 150 mM, KCl 5 mM, glucose 10 mM, HEPES 10 mM) (pH 7.4) and containing 1 mM EGTA, and the mixture was centrifuged at 150×*g* for 10 min. After the supernatant was further centrifuged at 400 × *g* for 5 min, the obtained pellet was suspended with 40 ml of the above Tyrode-Hepes solution and then further centrifuged at 400 × *g* for 5 min. The pellet was suspended with 2 ml of Ca^2+^-free Tyrode solution (NaCl 150 mM, KCl 5 mM, MgCl_2_ 1 mM, glucose 10 mM, and HEPES 10 mM) (pH 7.4), and the resulting platelet suspension was used for the experiments within 2 h after blood collection. The concentration of platelets in its suspension used for each experiment was adjusted to be approximately 10^5^/μl.

### Measurement of [Ca^2+^]_i_

[Ca^2+^]_i_ was ascertained using the fluorescent Ca^2+^ indicator Fura-2. Fura-2 is a ratiometric fluorescent indicator dye that binds to free intracellular calcium. Fura-2-acetoxymethyl ester (Fura-2/AM) is a membrane-permeant derivative of Fura-2 and is often used for measuring intracellular calcium. The washed platelets were loaded with Fura-2/AM (final concentration, 5 μM) at 37 °C for 30 min. After loading, the platelets were washed once with Ca^2+^- and Mg^2+^-free Tyrode solution buffered by Hepes (NaCl 150 mM, KCl 5 mM, glucose 10 mM, HEPES 10 mM) (pH 7.4) containing 1 mM EGTA, and they were resuspended in 2 ml of a Ca^2+^-free Tyrode solution buffered by Hepes (NaCl 150 mM, KCl 5 mM, MgCl_2_ 1 mM, glucose 10 mM, and HEPES 10 mM) (pH 7.4) (nominally Ca^2+^-free solution).

Fluorescence measurements were carried out with a dual-wavelength spectrofluorimeter (F-2500 Fluorescence Spectrophotometer, Hitachi High-Technologies Corporation, Tokyo, Japan) using a 0.4-ml cuvette maintained at 37 °C. The wavelengths used for excitation were 340 and 380 nm, and the wavelength used for emission was 510 nm. The fractional changes in [Ca^2+^]_i_ were determined using a ratio (R) of fluorescence intensity (F) of F340/F380. The fluorescence after sequential addition of 0.25% Triton X-100 and EGTA (5 mM) to the platelet suspension provided the maximum fluorescence ratio (Rmax) and minimum fluorescence ratio (Rmin), respectively. [Ca^2+^]_i_ was calculated using the following formula [11]:

$$ {\left[{\mathrm{Ca}}^{2+}\right]}_{\mathrm{i}}=\left(R-\mathrm{Rmin}\right)/\left(\mathrm{Rmax}-R\right)\times \beta \times \mathrm{Kd}, $$

where *β* is the ratio of the emission fluorescence values at 380-nm excitation in the presence of Triton X-100 and EGTA and Kd; the dissociation constant for Ca^2+^ is 224. Ca^2+^ entry induced by thrombin, thapsigargin, or OAG was expressed as the net increase in [Ca^2+^]_i_ calculated by subtraction of the basal [Ca^2+^]_i_ level from the maximum [Ca^2+^]_i_ level after stimulation.

### Measurement of platelet aggregation

Platelet aggregation was measured using platelets suspended in Ca^2+^-free Tyrode solution buffered by Hepes (NaCl 150 mM, KCl 5 mM, MgCl_2_ 1 mM, glucose 10 mM, and HEPES 10 mM) (pH 7.4) and evaluated using an aggregometer (IMI PRP313M, TAIYO Instruments INC., Osaka, Japan) that measures increases in light transmission through a cuvette (0.2 ml) containing stirred platelet suspension. The light transmission through a washed platelet suspension without any treatment and that through a suspended buffer not containing platelets were considered as 0 and 100%, respectively. The percentage of aggregation during the course of each experiment was calculated. The experimental conditions were the same as those for [Ca^2+^]_i_ measurement except for the volume of the cuvettes.

### Protocols for experiments assessing Ca^2+^ entry and platelet aggregation

Platelets were stabilized in a nominally Ca^2+^-free medium in the cuvette for 3 min and then stimulated with each stimulant (thrombin [0.025 U/ml], thapsigargin [1 µM], or OAG [100 μM]). At 1 min after the addition of each stimulant, the platelets were pretreated with methanol or formic acid at each concentration or a vehicle (distilled water) for 1 min. Then, Ca^2+^ entry and aggregation were induced by adding CaCl_2_ (0.5 mM) to the cuvette. It is known that threshold limit value (TLV) for methanol in the industry is 200 ppm, and exposure to this concentration of methanol causes blood methanol concentration of about 10 mg/L (0.00126%) [12]. In previous studies on acute methanol intoxication, blood concentrations of methanol were reportedly 147.3 mg/dl [13] and 1.75 g/l [14], which correspond to about 0.186 and 0.221%, respectively. Thus, blood concentrations of methanol in its acute intoxication are expected to be about 0.2%. Then, we first investigated the effects of these environmental and toxic concentrations of methanol (0.001, 0.02, and 0.2%) on platelet function. Higher pharmacological concentrations of methanol (0.5–2%) were also tested in further experiments. TLV of formic acid is known to be 5 ppm, and the blood concentrations of formic acid in patients with acute methanol poisoning who needed to be treated by dialysis therapy were reported to be 13.4 mg/dl [15] or 16.9 mg/dl [16], which correspond to about 0.0110 and 0.0139%, respectively. Then, we used concentrations of formic acid at 0.005–0.02% in this study.

### Drugs

Methanol (chemical-grade quality, 99.8 wt% purity) and formic acid (chemical-grade quality, 98.0 wt% purity) purchased from Wako Pure Chemical Co., Osaka, Japan, were prepared at the time of use and diluted with distilled water. Thapsigargin (Sigma, St Louis, Missouri, USA), OAG (Sigma), and Fura-2/AM (Dojindo Laboratories, Kumamoto, Japan) were dissolved in dimethylsulfoxide to make stock solutions of 1, 100, and 5 mM, respectively, and stored at − 80 °C. Bovine thrombin (Wako Pure Chemical) was dissolved in distilled water to make a stock solution of 1 U/μl and was stored at − 80 °C.

### Statistical analysis

The data are presented as means ± standard deviations. Statistical analysis was performed using the analysis of variance (ANOVA) followed by Scheffé *F* test. *p* values less than 0.05 were regarded as significant.

## Results

### Effects of methanol at environmental and toxic concentrations on platelet aggregation and Ca^2+^ entry into platelets induced by different stimuli

**Fig. 1:**
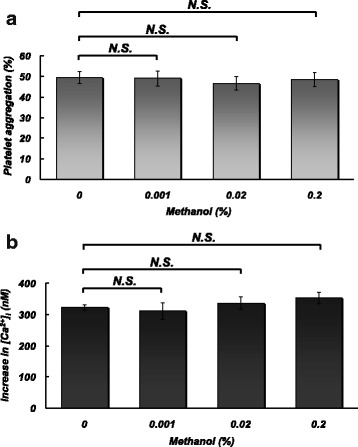
Effects of pretreatment with different concentrations (0, 0.001, 0.02, and 0.2%) of methanol on platelet aggregation (at 5 min) (**a**) and Ca^2+^ entry (**b**) induced by extracellular addition of CaCl_2_ (0.5 mM) in the presence of thrombin (0.025 U/ml). *N.S.* no significant difference. *N* = 4

Figure 1 shows the effects of methanol at 0.001, 0.02, and 0.2% on platelet aggregation (a) and Ca^2+^ entry into platelets (b) induced by thrombin. Methanol at these environmental and toxic concentrations significantly affected neither platelet aggregation nor Ca^2+^ entry into platelets. Platelet aggregation and Ca^2+^ entry into platelets induced by thapsigargin and OAG were also not significantly affected by methanol at 0.001, 0.02, and 0.2% (data not shown).

### Effects of methanol at pharmacological concentrations on platelet aggregation induced by different stimuli

**Fig. 2:**
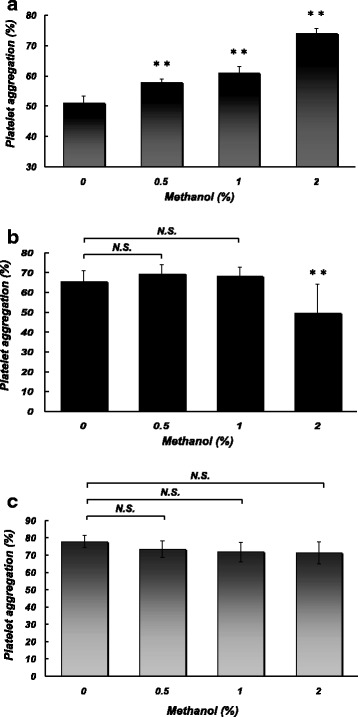
Effects of pretreatment with different concentrations (0, 0.5, 1, and 2%) of methanol on platelet aggregation (at 5 min) induced by extracellular addition of CaCl_2_ (0.5 mM) in the presence of thrombin (0.025 U/ml) (**a**), thapsigargin (1 μM) (**b**), or OAG (100 μM) (**c**). Asterisks denote significant differences (**p < 0.01) compared with the control. *N.S.* no significant difference. *N* = 5

Figure 2 shows the effects of methanol at 0.5–2% on platelet aggregation induced by thrombin (a), thapsigargin (b), and OAG (c). Thrombin-induced aggregation was significantly increased by methanol in a concentration-dependent manner. On the other hand, methanol at a higher concentration (2%) but not lower concentrations (0.5 and 1%) significantly attenuated thapsigargin-induced aggregation. OAG-induced aggregation was not significantly affected by methanol at 0.5–2%.

### Effects of methanol at pharmacological concentrations on Ca^2+^ entry into platelets induced by different stimuli

**Fig. 3:**
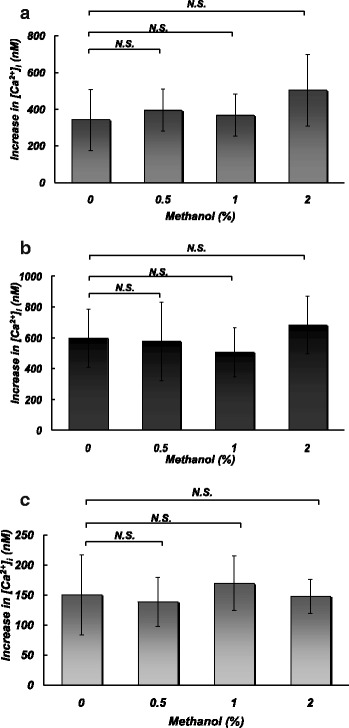
Effects of pretreatment with different concentrations (0, 0.5, 1, and 2%) of methanol on Ca^2+^ entry induced by extracellular addition of CaCl_2_ (0.5 mM) in the presence of thrombin (0.025 U/ml) (**a**), thapsigargin (1 μM) (**b**), or OAG (100 μM) (**c**). *N.S.* no significant difference. *N* = 6–8

Figure 3 shows the effects of methanol at 0.5–2% on Ca^2+^ entry into platelets corresponding to their aggregation induced by thrombin (a), thapsigargin (b), and OAG (c). Methanol at the above concentrations did not significantly affect Ca^2+^ entry into platelets induced by any of the three stimulants.

### Effects of formic acid at toxic concentrations on platelet aggregation induced by different stimuli

**Fig. 4:**
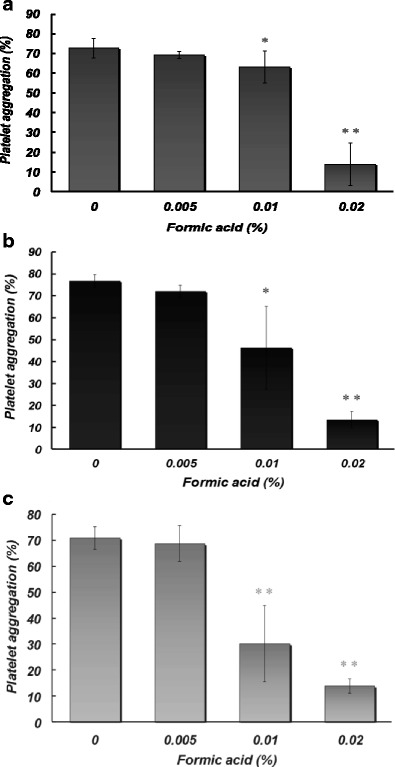
Effects of pretreatment with different concentrations (0, 0.005, 0.01, and 0.02%) of formic acid on platelet aggregation (at 5 min) induced by extracellular addition of CaCl_2_ (0.5 mM) in the presence of thrombin (0.025 U/ml) (**a**), thapsigargin (1 μM) (**b**), or OAG (100 μM) (**c**). Asterisks denote significant differences (*p < 0.05; **p < 0.01) compared with the control. *N* = 4–10

Figure 4 shows the effects of formic acid at 0.005–0.02% on platelet aggregation induced by thrombin (a), thapsigargin (b), and OAG (c). Platelet aggregation induced by thrombin, thapsigargin, or OAG was significantly attenuated by formic acid at 0.01 and 0.02%.

### Effects of formic acid at toxic concentrations on Ca^2+^ entry into platelets induced by different stimuli

**Fig. 5:**
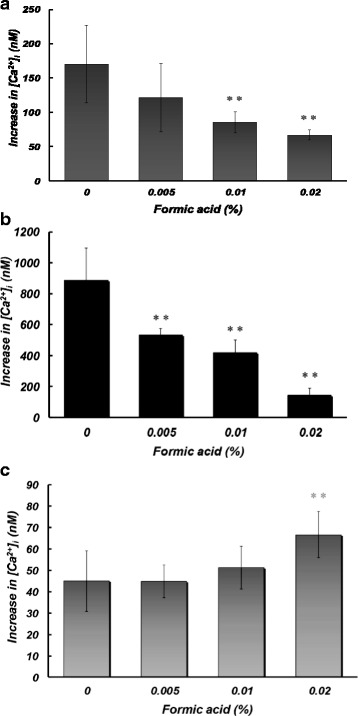
Effects of pretreatment with different concentrations (0, 0.005, 0.01, and 0.02%) of formic acid on Ca^2+^ entry induced by extracellular addition of CaCl_2_ (0.5 mM) in the presence of thrombin (0.025 U/ml) (**a**), thapsigargin (1 μM) (**b**), or OAG (100 μM) (**c**). Asterisks denote significant differences (**p < 0.01) compared with the control. *N* = 8–9

Figure 5 shows the effects of formic acid at 0.005–0.02% on Ca^2+^ entry into platelets corresponding to their aggregation induced by thrombin (a), thapsigargin (b), and OAG (c). Formic acid at 0.01 and 0.02% significantly inhibited Ca^2+^ entry into platelets induced by thrombin and thapsigargin. Formic acid at 0.005% also significantly reduced Ca^2+^ entry into platelets induced by thapsigargin. On the other hand, formic acid at a higher concentration of 0.02% but not at 0.005 and 0.01% significantly augmented Ca^2+^ entry into platelets induced by OAG.

### Effects of formic acid on extraplatelet pH

Additions of formic acid at 0.005, 0.01, and 0.02% to the solution suspending platelets lowered its pH (means ± standard deviations) from 7.424 ± 0.005 to 7.299 ± 0.043 (*p* < 0.05), 7.017 ± 0.013 (*p* < 0.01), and 6.234 ± 0.085 (*p* < 0.01), respectively.

## Discussion

Although environmental and toxic concentrations of methanol did not affect platelet aggregation and Ca^2+^ entry into platelets, methanol at pharmacological doses showed diverse effects on platelet aggregation that depended on the aggregating stimulant. Thrombin-induced aggregation was increased by methanol (0.5–2%), while thapsigargin-induced aggregation was inhibited by a higher concentration (2%) of methanol but was not significantly changed by lower concentrations. Platelet aggregation is known to depend strongly on Ca^2+^ entry [9], and thus we suspected that Ca^2+^ entry pathways would be affected by methanol. In this study, we used three platelet activating stimulants that act through different mechanisms. Thrombin activates its receptors, resulting in hydrolysis of phosphoinositides and subsequent production of two signal transduction messengers, inositol trisphosphate and diacylglycerol. The former induces mobilization of Ca^2+^ from its intracellular stores, which triggers transmembranous Ca^2+^ entry, so-called capacitative Ca^2+^ entry, through TRPC (transient receptor potential canonical) 1/4/5 channels [17]. The latter facilitates TRPC3/6/7 channel-mediated Ca^2+^ entry [18, 19]. Thapsigargin and OAG were used as mimics of inositol trisphosphate and diacylglycerol, respectively. However, methanol did not affect Ca^2+^ entry induced by any of the stimulants used in this study. Therefore, the effects of methanol on platelet aggregation are thought to be independent of Ca^2+^ entry, and methanol is suggested to affect signaling pathways in platelets that are downstream of the increase in intracellular Ca^2+^ concentrations. Further studies are needed to clarify the mechanisms for the diverse effects of methanol on platelet aggregation induced by thrombin and thapsigargin.

Blood concentrations of methanol in its acute intoxication have been reported to be about 0.2% [13, 14]. Thus, the concentrations of methanol that exerted significant actions on platelet aggregation in the present study were higher than those attained under an environmental condition and those observed in patients with acute methanol intoxication. Therefore, it is unlikely that methanol influences blood coagulability in vivo through its direct action on platelet aggregation even under a toxic condition.

On the other hand, formic acid, the final metabolite of methanol in vivo, at toxic concentrations showed inhibitory effects on platelet aggregation and corresponding Ca^2+^ entry in platelets in response to thrombin and thapsigargin. Therefore, formic acid inhibits platelet aggregation through attenuating capacitative Ca^2+^ entry. Since thrombin is a physiological agonist for platelet aggregation, there is a possibility that bleeding tendency due to attenuation of platelet aggregation occurs in patients with methanol poisoning, and further clinical studies are needed to test this hypothesis. Platelet aggregation induced by OAG was also attenuated in the presence of formic acid, while corresponding Ca^2+^ entry in platelets induced by OAG was not affected by formic acid at lower concentrations (0.005 and 0.01%) and was rather augmented by formic acid at a higher concentration (0.02%). Thus, in addition to the inhibitory action on capacitative Ca^2+^ entry, there may be another inhibitory mechanism, besides action on Ca^2+^ entry, for formic acid-induced inhibition of platelet aggregation. This is the first study showing the inhibitory action of formic acid on Ca^2+^ entry into platelets and subsequent aggregation of them. Further studies are also needed to clarify the Ca^2+^ entry-independent pathway for platelet aggregation that is inhibited by formic acid.

The pH of the solution suspending platelets was lowered by the addition of formic acid (0.005–0.02%) in a concentration-dependent manner. We previously reported that thapsigargin-induced Ca^2+^ entry into and subsequent aggregation of platelets were decreased under the condition of extracellular acidosis (pH 6.9) and were increased under the condition of extracellular alkalosis (pH 7.9) [20]. Thus, platelet aggregation induced by thapsigargin depends on extracellular pH in the range of pH from 6.9 to 7.9. Interestingly, the degrees of inhibition of Ca^2+^ entry and subsequent aggregation by extracellular acidosis (pH 6.9) [20] were comparable to the degrees of inhibition by formic acid at 0.01% (Figs. 4 and 5), which caused a similar (slightly lower) degree of intracellular acidosis (pH 7.017 ± 0.013) in the present study. Therefore, we concluded that formic acid-induced inhibition of Ca^2+^ entry and subsequent aggregation resulted mainly from extracellular acidosis induced by formic acid. This explanation is plausible because metabolic acidosis occurs in patients with severe methanol poisoning [21].

**Fig. 6:**
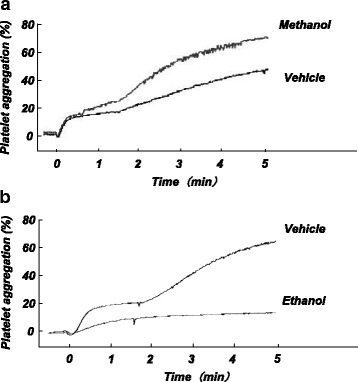
Representative recordings of thrombin-induced platelet aggregation in the presence or absence of methanol (**a**) or ethanol (**b**). The effect of ethanol on platelet aggregation was examined by the same method as that used in the present study. Methanol (2%), ethanol (2%), or a vehicle was added to a platelet suspension in the nominally Ca^2+^-free solution. Platelets were stimulated with thrombin (0.025 U/ml), and platelet aggregation was induced by extracellular addition of CaCl_2_ (0.5 mM) at time 0 and was measured by the light transmission method as mentioned in the “Methods” section

Table 1 compares the effects of methanol and ethanol on Ca^2+^ entry and subsequent platelet aggregation. In our previous study, ethanol at its toxic concentrations (0.5–2%) inhibited platelet aggregation in a concentration-dependent manner irrespective of platelet stimuli [10]. The effects of ethanol on Ca^2+^ entry were complicated. Ethanol decreased Ca^2+^ entry induced by thapsigargin and increased that induced by OAG and did not significantly affect that induced by thrombin [10]. Thus, ethanol has diverse actions on Ca^2+^ entry mediated by TRPC1/4/5 and TRPC3/6/7 channels, and these actions were thought to be canceled when platelets were stimulated with thrombin, which activates both TRPC1/4/5 and TRPC3/6/7 channels. In addition to these actions on Ca^2+^ entry, ethanol has an inhibitory effect on platelet aggregation independent of Ca^2+^ entry. Thus, the effects of ethanol on Ca^2+^ entry into and aggregation of platelets are quite different from the effects of methanol reported in the present study. Figure 6 shows representative recordings of thrombin-induced platelet aggregation in the presence of the same concentration (2%) of methanol and ethanol, which increased and decreased the aggregation, respectively. Since both methanol and ethanol are membrane-permeable, they are speculated to affect not only different types of Ca^2+^ channels in the plasmalemma but also various intracellular organelles. Further studies are needed to determine the reasons for the differing actions of methanol and ethanol on Ca^2+^ entry into and aggregation of platelets, and to clarify how these actions are related to the differences in the structures of these alcohol variants.

**Table 1:** Comparison of effects of methanol and ethanol on aggregation of and Ca^2+^ influx into platelets induced by different stimulants

	Methanol	Ethanol
Aggregation		
Thrombin	↑	↓
Thapsigargin	→↓	↓
OAG	→	↓
Ca influx		
Thrombin	→	→
Thapsigargin	→	↓
OAG	→	↑

↑ increase; ↓ decrease; → no change

## Conclusion

Methanol, at pharmacological doses (0.5% or higher) but not at toxic doses, had diverse actions on platelet aggregation—levels of aggregation induced by thrombin and thapsigargin were increased and decreased, respectively, by methanol. On the other hand, methanol showed no significant effects on Ca^2+^ entry in platelets. Thus, methanol has diverse effects on platelet aggregation, depending on the aggregation stimuli, without affecting Ca^2+^ entry into platelets. Formic acid at toxic doses (0.01% or higher) showed significant inhibitory actions on platelet aggregation and corresponding Ca^2+^ entry in platelets, which may be caused by extracellular acidosis. Therefore, it is suggested that, in methanol intoxication, platelet aggregation is not affected by methanol itself but is inhibited by its metabolite, formic acid, resulting in bleeding tendency, which may be involved in etiology of complicated brain hemorrhage [8].

## Abbreviations

ANOVA: Analysis of variance
Fura-2/AM: Fura-2-acetoxymethyl ester
OAG: 1-oleoyl-2-acetyl-sn-glycerol
PRP: Platelet-rich plasma
Rmax: Maximum fluorescence ratio
Rmin: Minimum fluorescence ratio
TLV: Threshold limit value
TRPC: Transient receptor potential canonical
